# Supplementary material for: Are replication rates the same across academic fields? Community forecasts from the DARPA SCORE programme
Source: R Soc Open Sci. 2020 Jul 22;7(7):200566. doi: 10.1098/rsos.200566 (PMC7428244; doi:10.1098/rsos.200566)
Supplement: Electronic supplementary material [file rsos200566supp1.pdf]

Are replication rates the same across academic fields? Community forecasts from the DARPA SCORE program

Michael Gordon[1,†], Domenico Viganola[2,†], Michael Bishop[3], Yiling Chen[4], Anna Dreber[5,6], Brandon Goldfedder[7], Felix Holzmeister[6], Magnus Johannesson[5], Yang Liu[8], Charles Twardy[9,10], Juntao Wang[4], Thomas Pfeiffer[1]

[†]The two first authors contributed equally to this work.

[1]New Zealand Institute for Advanced Study, Massey University, Auckland, New Zealand; [2]Department of Systems Engineering and Operations Research, George Mason University, Fairfax, VA, USA; [3]Michael Bishop Consulting, Ottawa, Canada; [4]John A. Paulson School of Engineering and Applied Sciences, Harvard University, Cambridge, MA, USA; [5]Department of Economics, Stockholm School of Economics, Stockholm, Sweden; [6]Department of Economics, University of Innsbruck, Innsbruck, Austria; [7]Gold Brand Software, LLC, Herndon, VA, USA; [8]Department of Computer Science and Engineering, University of California, Santa Cruz, CA, USA; [9]Jacobs Engineering Group Inc., Herndon, VA, USA; [10]C4I & Cyber Center, George Mason University, Fairfax, VA, USA

Supplementary Material

Table S1. List of Journals and Discipline Clusters

Journal	Discipline Cluster
**American Economic Journal: Applied Economics**	Economics
**American Economic Review**	Economics
**Econometrica**	Economics
**Experimental Economics**	Economics
**Journal of Finance**	Economics
**Journal of Financial Economics**	Economics
**Journal of Labor Economics**	Economics
**Journal of Political Economy**	Economics
**Quarterly Journal of Economics**	Economics
**Review of Financial Studies**	Economics
**American Educational Research Journal**	Education
**Computers and Education**	Education
**Contemporary Educational Psychology**	Education
**Educational Researcher**	Education
**Exceptional Children**	Education
**Journal of Educational Psychology**	Education
**Learning and Instruction**	Education
**Academy of Management Journal**	Marketing, Management and Related Areas
**Journal of Business Research**	Marketing, Management and Related Areas
**Journal of Consumer Research**	Marketing, Management and Related Areas
**Journal of Management**	Marketing, Management and Related Areas
**Journal of Marketing**	Marketing, Management and Related Areas
**Journal of Marketing Research**	Marketing, Management and Related Areas
**Journal of Organizational Behavior**	Marketing, Management and Related Areas
**Journal of Public Administration Research and Theory**	Marketing, Management and Related Areas
**Journal of the Academy of Marketing Science**	Marketing, Management and Related Areas
**Leadership Quarterly**	Marketing, Management and Related Areas
**Management Science**	Marketing, Management and Related Areas
**Organization Science**	Marketing, Management and Related Areas
**Organizational Behavior and Human Decision Processes**	Marketing, Management and Related Areas

**Public Administration Review**	Marketing, Management and Related Areas
**American Journal of Political Science**	Political Science
**American Political Science Review**	Political Science
**British Journal of Political Science**	Political Science
**Comparative Political Studies**	Political Science
**Journal of Conflict Resolution**	Political Science
**Journal of Experimental Political Science**	Political Science
**World Development**	Political Science
**World Politics**	Political Science
**Child Development**	Psychology
**Clinical Psychological Science**	Psychology
**Cognition**	Psychology
**European Journal of Personality**	Psychology
**Evolution and Human Behavior**	Psychology
**Health Psychology**	Psychology
**Journal of Applied Psychology**	Psychology
**Journal of Consulting and Clinical Psychology**	Psychology
**Journal of Environmental Psychology**	Psychology
**Journal of Experimental Psychology: General**	Psychology
**Journal of Experimental Social Psychology**	Psychology
**Journal of Personality and Social Psychology**	Psychology
**Psychological Medicine**	Psychology
**Psychological Science**	Psychology
**American Journal of Sociology**	Sociology & Criminology
**American Sociological Review**	Sociology & Criminology
**Criminology**	Sociology & Criminology
**Demography**	Sociology & Criminology
**European Sociological Review**	Sociology & Criminology
**Journal of Marriage and Family**	Sociology & Criminology
**Law and Human Behavior**	Sociology & Criminology
**Social Forces**	Sociology & Criminology
**Social Science and Medicine**	Sociology & Criminology

Table S2. List of questions in the initial survey and market

Q1) What will be the average replication rate in SCORE?
Q2) What will be the average replication rate in economics?
Q3) What will be the average replication rate in political sciences?
Q4) What will be the average replication rate in psychology?
Q5) What will be the average replication rate in education research?
Q6) What will be the average replication rate in sociology and criminology?
Q7) What will be the average replication rate in marketing, management and related areas?
Q8) What will be the average replication rate for papers published in 2009/10?
Q9) What will be the average replication rate for papers published in 2011/12?
Q10) What will be the average replication rate for papers published in 2013/14?
Q11) What will be the average replication rate for papers published in 2015/16?
Q12) What will be the average replication rate for papers published in 2017/18?

Table S3. *p*-values for pairwise *t*-tests for time-specific responses (df = 225 for all tests)

	**2009/10**	**2011/12**	**2013/2014**	**2015/2016**
**2011/2012**	t = 2.741, p = 0.00662			
**2013/2014**	t = 10.6641, p < 0.00001	t = 6.3453, p < 0.00001		
**2015/2016**	t = 9.7299, p < 0.00001	t = 13.7561, p < 0.00001	t = 5.0903, p < 0.00001	
**2017/2018**	t = 14.9263, p < 0.00001	t = 13.6037, p < 0.00001	t =14.6781, p < 0.00001	t =8.9061, p < 0.00001

Table S4. *p*-values for pairwise *t*-tests for topic-specific responses (df = 225 for all tests)

	**Economics**	**Education**	**Marketing & Management**	**Political Science**	**Psychology**
**Education**	t = -14.4446, p < 0.00001				
**Marketing & Management**	t = -12.866, p < 0.00001	t = 1.8142, p = 0.08190			
**Political Science**	t = -11.6888, p < 0.00001	t = 6.8161, p < 0.00001	t = 5.0736, p < 0.00001		
**Psychology**	t = -16.202, p < 0.00001	t = 0.4283, p = 0.6688	t = -1.2820, p = 0.2155	t = -7.3937, p < 0.00001	
**Sociology & criminology**	t = -11.5689, p < 0.00001	t = 4.3917, p = 0.00003	t = 1.8982, p = 0.07368	t = -3.9677, p = 0.00015	t = 3.2547, p = 0.00179

Table S5. Relation between forecast for the overall replication rate in score and demographic characteristics. The reference category for career stage is ‘student’; category ‘other’ includes those who did not provide an answer as well as those who chose ‘prefer not to answer’ and ‘other’. ‘Academia’ indicates those participants currently involved in academic activities either as a student or an employee. ‘Prediction market’ refers to participants who had been involved in a previous prediction markets. ‘Replication’ indicates that the participants have been involved in a replication study before. There is suggestive evidence that having being involved in a replication study reduces the response for the overall SCORE replication rate forecast. An ANOVA indicates that the career stage variable has no statistically significant joined effect on the forecast ($F_4 = 0.396$, $p = 0.8115$).

Term	Estimate	SE	Statistic	*p*-value
**Intercept**	51.0722	5.5696	9.1697	> 0.00001
**Career stage: early career**	0.9058	2.4419	0.3709	0.7110
**Career stage: mid career**	2.3884	2.7892	0.8563	0.3928
**Career stage: other**	-3.5457	5.1816	-0.6843	0.4945
**Career stage: senior career**	0.09366	3.6697	0.02552	0.9797
**Academia**	-1.0654	5.2513	-0.2029	0.8394
**Prediction market**	2.1015	1.8282	1.1495	0.2516
**Replication**	-5.2153	1.8943	-2.7531	0.0064
